# Estimation of Characteristic Parameters of Grape Clusters Based on Point Cloud Data

**DOI:** 10.3389/fpls.2022.885167

**Published:** 2022-07-14

**Authors:** Wentao Liu, Chenglin Wang, De Yan, Weilin Chen, Lufeng Luo

**Affiliations:** School of Mechatronics Engineering and Automation, Foshan University, Foshan, China

**Keywords:** grapes, point cloud, feature parameter, volume, Poisson reconstruction

## Abstract

The measurement of grapevine phenotypic parameters is crucial to quantify crop traits. However, individual differences in grape bunches pose challenges in accurately measuring their characteristic parameters. Hence, this study explores a method for estimating grape feature parameters based on point cloud information: segment the grape point cloud by filtering and region growing algorithm, and register the complete grape point cloud model by the improved iterative closest point algorithm. After estimating model phenotypic size characteristics, the grape bunch surface was reconstructed using the Poisson algorithm. Through the comparative analysis with the existing four methods (geometric model, 3D convex hull, 3D alpha-shape, and voxel-based), the estimation results of the algorithm proposed in this study are the closest to the measured parameters. Experimental data show that the coefficient of determination (*R*^2^) of the Poisson reconstruction algorithm is 0.9915, which is 0.2306 higher than the coefficient estimated by the existing alpha-shape algorithm (*R*^2^ = 0.7609). Therefore, the method proposed in this study provides a strong basis for the quantification of grape traits.

## Introduction

Quantifying plant traits is critical to the development of precision agriculture (Comba et al., 2015, 2018). This minimizes costs and maximizes profitability. The detection of plant parameters is of great significance for understanding their growth status, improving their quality, improving breeding methods, and evaluating yield (Tourneux et al., 2003; Schmidt et al., 2014). Using computer vision technology to detect various parameters of plants has been proven to be an effective means of analyzing features. Many researchers have carried out experiments based on the three aspects of phenotypic character detection, maturity detection, and nutritional content detection of this technology (Füzy et al., 2019). Weight, size, and volume are important phenotypic parameters. They can be used not only as an indicator of plant growth vigor but also as a parameter for estimating traits (Wang and Chen, 2020a; Zevgolis et al., 2021). Table grapes are plants with variable spatial structures and complex geometric shapes. Relevant studies have shown that their phenotypic traits (such as weight, width, length, and the number of grapes on a bunch) are significantly related to grape growing environment, composition (such as sweetness and acidity), and heritability (Sato et al., 2000; Fanizza et al., 2005; Liu et al., 2007). Therefore, this paper takes grape bunches as the research object, focusing on the detection of phenotypic parameters of grapes.

Most of the traditional non-contact measurement research is based on two-dimensional image processing (Luo et al., 2021). This method is mainly used in the detection of phenotypic parameters of uncomplicated fruits, such as apples, apricots, and red dates. They objectively describe fruit size by extracting their texture (Muhammad, 2014), color (Khojastehnazhand et al., 2019), shape (Wu et al., 2019), and other characteristics. The above studies can detect and classify fruits and vegetables. However, due to the lack of information in the collected 2D data where the plant is occluded (including self-occlusion), missing parts cannot be recovered by hypothetical fit. Therefore, it is difficult to measure complex fruits accurately.

In recent years, the high-precision and high-resolution sampling of 3D laser equipment has provided the possibility of measurement work (Paulus et al., 2014; Xiang et al., 2019). Various methods of reconstruction from 3D data have been proposed. Remote sensing techniques are used in some studies to process collected ground information for reconstruction (Moreno et al., 2020). For example, vineyard yield can be predicted by segmenting features of local areas or isolating elements of interest (Delenne et al., 2010; López-Granados et al., 2020). These are mainly used in the management of botanical gardens. Due to the complexity of plant internal structures, accurate reconstruction of the complete 3D structure is more successful in determining the detailed phenotypic parameters of plants. Accurate reconstruction of 3D models of plants has always been one of the focuses of computer graphics and agronomy research (Wang and Chen, 2020b).

In general, grape models can be rendered realistically using a rule-based approach. Huang et al. describe a grape modeling method based on Open L-system (Huang et al., 2013). The best fit hypothesis is generated as the final reconstruction result by the grape parameters set by the user. Ivorra et al. (2015) proposed a 3D computer vision method that automatically generates grape models based on new 3D descriptors. After obtaining partial 3D information, an SVM model based on new 3D descriptors is used to predict the invisible grape components. However, the above is only a hypothetical model for visualization purposes. In 2015, Schöler and Steinhage (2015) proposed a method for reconstructing grape bunch structures from full point clouds. The topology and geometry of the grape point cloud are obtained by applying random sample consensus, and the entire model is then optimized by modifying the parameter values and the number of components. It focuses on modeling the interconnectivity of regions of interest. In 2017, Mack et al. (2017) applied the area growing method to group the identified grapes into coherent patches on the bunch surface. The grapes are generated by applying the patches and the structure of the grape stem is reconstructed from the generated grapes. Their goal is not to accurately estimate the characteristic parameters of grape bunches but only to produce a plausible model, especially for low-density grape bunches. Some scholars have explored different methods to explicitly restore the plant surface to automatically estimate parameters such as plant volume and size. This estimate can be replaced with approximate shapes derived from pixel area calculations (Wang et al., 2017) or stereo methods (Gongal et al., 2018; Yan et al., 2019; You et al., 2019).

Near-realistic grape models cannot be generated due to the tight occlusion of grapes and the partial measurements of grapes within the bunch. In addition, there is currently no method based on explicit reconstruction to restore the pores and crevices inside grape bunches. Based on the point cloud collected from multiple angles, this study proposes a grape model reconstruction algorithm, which combines the improved iterative closest point (ICP) algorithm with Poisson reconstruction. The proposed model and four existing models are tested, including a consideration of the relationship between volume and true value for different algorithms. In the context of estimating grape phenotypic parameters, we can accurately match the point cloud and reconstruct the details of the grape model from the point cloud.

## Data Acquisition

**Figure 1:**
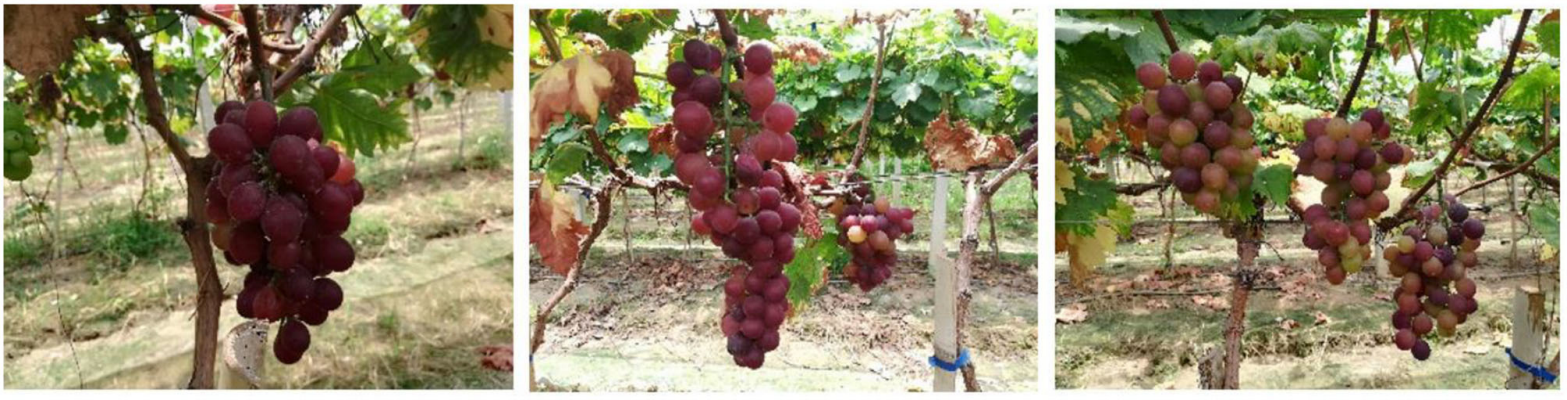
Red grape map.

**Figure 2:**
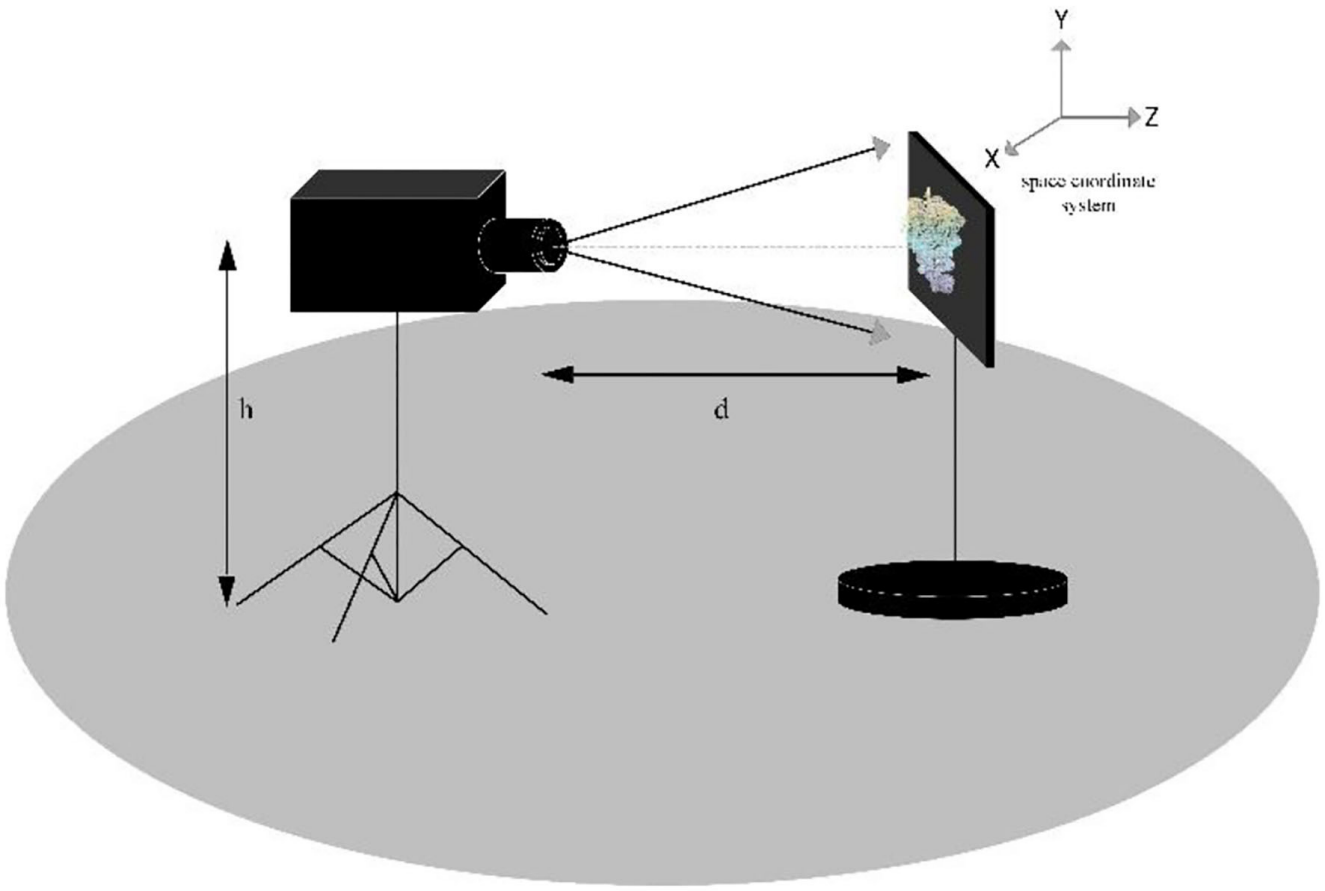
Diagram of the location of the experimental equipment in the grape characteristic parameter measurement system.

The red grape is a common grape type, with large and relatively space-filling grapes. The shape of the bunch is similar to a cylinder, as shown in Figure 1. This study used 16 randomly selected red grape samples of different shapes and sizes on different vines. The selected bunches are set in front of the positioning plate, with the vertical height h of the point cloud camera set as 0.75 m and the distance d from the grapes set as 1 m. The space coordinate system is set as follows: the direction of the main axis passing through the camera center point to the grape bunch is the Z-axis, the vertical axis is the Y-axis, and the X-axis is the remaining horizontal axis. Figure 2 shows the schematic diagram of the experimental equipment of the grape characteristic parameter measurement system. The point cloud camera system collects and saves the point cloud data of Hongti grapes from different perspectives. The 3D point cloud system consists of a point cloud camera (kinectv2, the accuracy error of the point cloud camera is 2 mm, and the range of capturing the point cloud is 0.5–4.5 m) and a microcomputer. The accuracy error of the point cloud camera is 2 mm, and the range of point cloud capture is 0.5–4.5 m, which is detailed enough to accurately model the grapes used in this study.

## Methods

### Point Cloud Preprocessing

**Figure 3:**
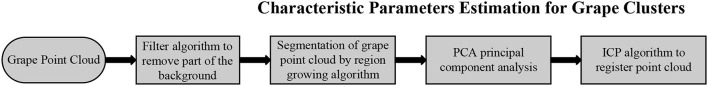
Grape point cloud data preprocessing flowchart.

The processing flow of grape point cloud data is shown in Figure 3. First, the point cloud scanning system is used to scan the point cloud information of the picked grapes. Next, to reduce the amount of data processing needed to generate the end model, the non-target point cloud area in the original point cloud is removed algorithmically, and the grape point cloud for the current camera perspective is obtained. The PCA algorithm is then used to obtain the coordinate system of the point cloud for each perspective, and the point cloud of each adjacent perspective is transformed rotationally through coordinate transformation to refine and confirm the accuracy of the model. Finally, the grape point clouds from different viewing angles are spliced together by the ICP algorithm to obtain a complete 3D model, which is convenient for subsequent representation information estimation and volume estimation.

#### Point Cloud Segmentation

**Figure 4:**
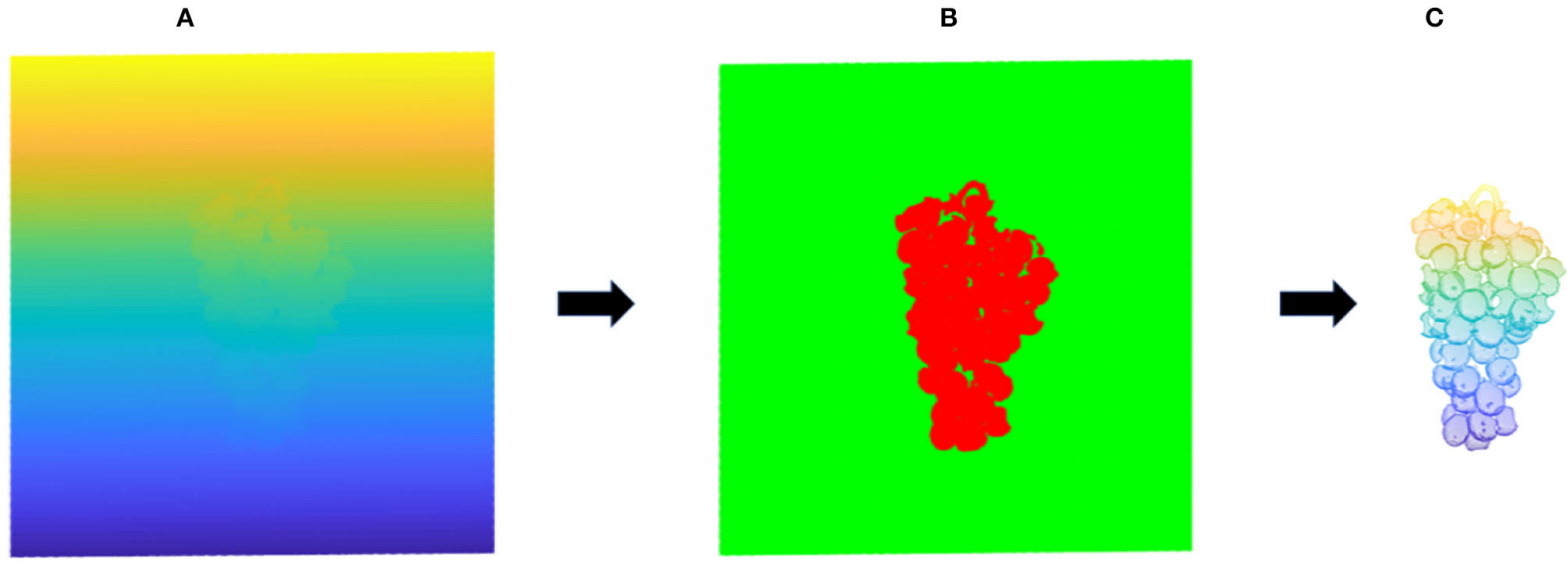
Point cloud segmentation map. **(A)** Represents the original point cloud image; **(B)** represents the clipping of outliers; **(C)** represents the grape point cloud extraction.

In this section, we will discuss how to segment the point cloud information of grapes from the original point cloud data. The raw point cloud data of a single view obtained by the camera scanning the grapes contain the target point cloud and the non-target point cloud. Segmentation algorithms such as Random Sample Consistency (RANSAC) and Region Growing are commonly used to extract target objects in the original point cloud. The grape bunch is not a simple geometric shape; it is similar to a complex convex irregular shape formed by stacking a large number of similar small spheres. The maturity of different grapes on the same bunch is different, which cannot be segmented by color features. To improve the efficiency of the target segmentation algorithm, some background point clouds can be removed by filtering the algorithm according to scene characteristics. First, the point cloud data of this experiment are collected by linear structured light. The grapes reflect this light in accordance with a convex structure. The distribution curvature along the z-axis changes greatly, while the distribution along the X- and Y-directions is in a limited range. According to this property, the point cloud can be clipped within a certain height range in the X-axis and Y-axis directions. Due to the large difference in curvature value between the normal of the grape point cloud and the normal of the background, the region growing algorithm was used to calculate its normal and curvature. Starting from the point with the smallest curvature, which is the seed point, the angle between the normal and curvature of each adjacent point and the normal and curvature of the current seed point can be calculated; if the angle is less than the threshold, add the current point into the seed until no points that satisfy these conditions can be found. Through this method, the non-target point cloud is filtered, and the point cloud information of the grapes is extracted, as shown in Figure 4.

#### Point Cloud Registration

**Figure 5:**
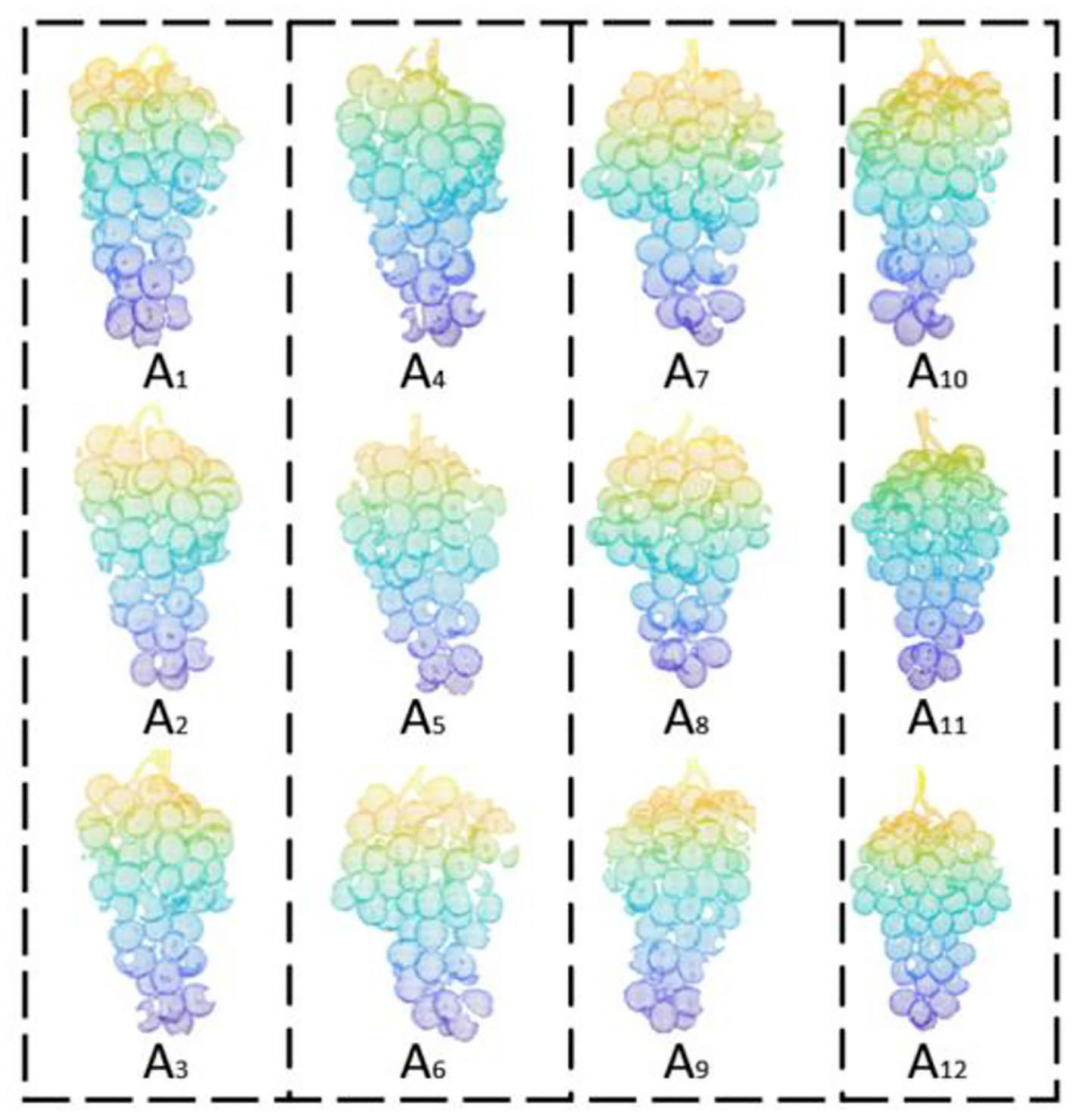
Point cloud renderings of a grape bunch from all angles.

Grapes overlap each other in an unpredictable way. In the actual collection process, because the overlapping grapes are blocked or the point cloud camera does not capture enough detail, the point cloud collected from a single angle will lead to the inability to obtain a complete grape model, so that its characteristic parameters cannot be accurately estimated. Therefore, to obtain a complete model, it is necessary to scan point clouds from different angles for registration. In this paper, the grapes are hung on the positioning plate, the point cloud of the grapes is scanned once, and then, the grapes are rotated counterclockwise around the Y-axis every 30° to obtain the grape point cloud from 12 different perspectives. This interval is sufficiently small to compare adjacent point clouds and reduce the impact of holes on the subsequent volume estimation accuracy. Figure 5 shows a point cloud rendering of a grape bunch collected from various angles.

In this paper, the principal component analysis (PCA) method is used to obtain the coordinate system of the point cloud blocks of each viewpoint. The point cloud blocks of adjacent viewing angles are transformed to relatively correct positions through coordinate transformation to achieve the purpose of rough matching. Then, the grape point cloud patches are registered from different angles using the iterative closest point (ICP) algorithm.

PCA preserves the main geometric information of grapes by mapping point cloud data from three-dimensional data to a set of uncorrelated two-dimensional data. PCA begins with the selection of the direction with the widest distribution of mapping points of the data as its first coordinate axis. In the plane orthogonal to the first coordinate axis, the second coordinate axis has the largest variance, and in the plane orthogonal to the first two coordinate axes, the third coordinate axis has the largest variance.

Solving the covariance of the point cloud {*A*_*i*_, *i* = 1, *n*} requires an n^*^3 matrix. After the mean value of point cloud A is normalized, the covariance matrix is solved as shown in Equation 1.


(1) $$\begin{array}{l} Cov\left(X,Y,Z\right)=\left[\begin{array}{ccc} Cov\left(x,x\right) & Cov\left(x,y\right) & Cov\left(x,z\right)\\ Cov\left(y,x\right) & Cov\left(y,y\right) & Cov\left(y,z\right)\\ Cov\left(z,x\right) & Cov\left(z,y\right) & Cov\left(z,z\right) \end{array}\right] \end{array}$$

Singular value decomposition (SVD) is used to decompose the covariance matrix *Cov* and solve the eigenvalues and eigenvectors, as shown in Equation 2. *U* is the eigenvector and Σ is the eigenvalue.


(2) $$\begin{array}{l} Cov\left(X,Y,Z\right)=U\Sigma V^{T} \end{array}$$

In the eigenvector, the eigenvector corresponding to the maximum eigenvalue is regarded as the Z-axis of the PCA coordinate system. The point set to be matched is then processed to realize rough matching.

**Figure 6:**
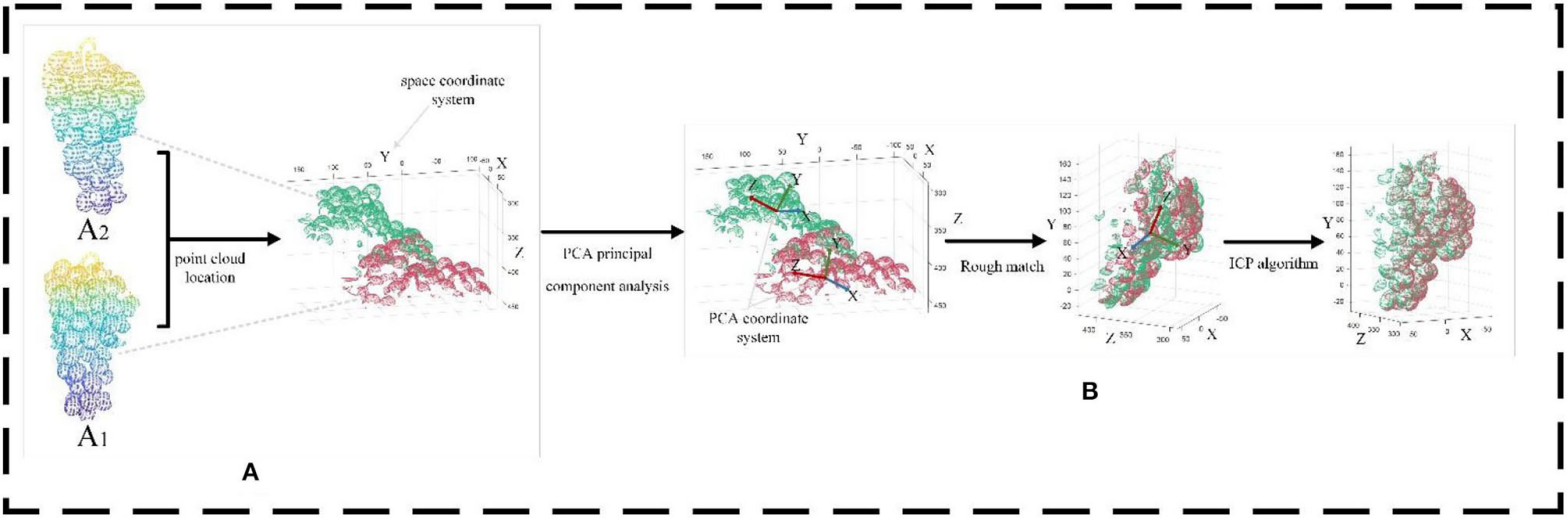
Matching results of the ICP algorithm and the ICP algorithm after applying the PCA algorithm are compared. **(A)** Red denotes the A_1_ point cloud, and green denotes the A_2_ point cloud. **(B)** Registration result of the improved ICP algorithm.

The main purpose of ICP is to take one of the point cloud sets as the source point set and the other point cloud set as the point set to be matched. After the sampling points in the point set to be matched are rotated and moved, the matrix R with the minimum error between the sampling points and the target point set is solved by the least square method. In this paper, the ICP algorithm is used for registration because a suitable registration effect can be obtained without the need for segmentation and feature extraction of the point cloud, as shown in Figure 6.

**Figure 7:**
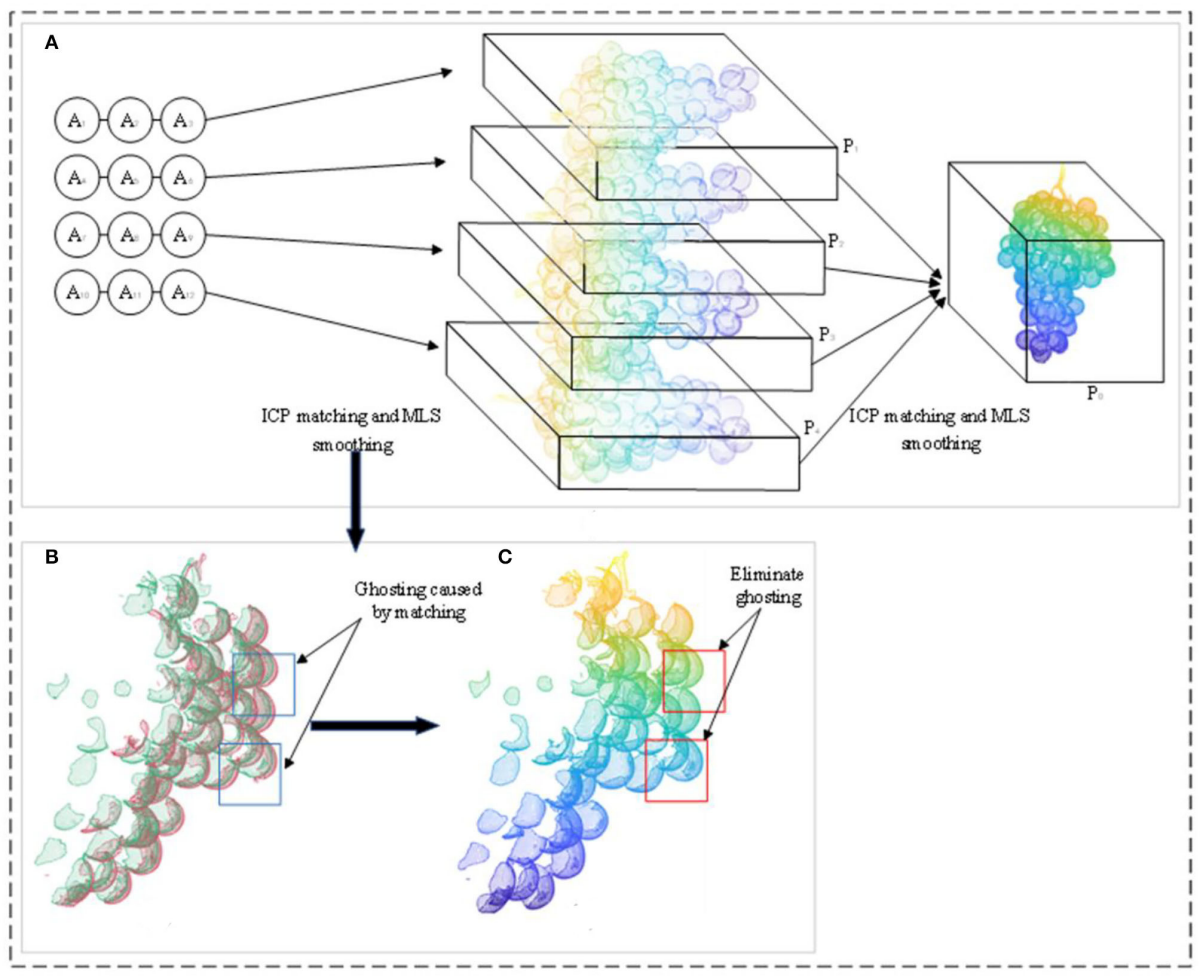
Point cloud data processing; **(A)** the step of obtaining the complete grape point cloud model P0; **(B)** the point cloud after the registration of the point cloud data of the two angles before smoothing, which produces ghost phenomena (blue boxes); **(C)** is the rendered image obtained after MLS smoothing in **(B)**, which eliminates the ghost phenomenon (red boxes).

Finally, A_1_, A_2_, and A_3_ are registered to generate Ā_1_. Similarly, Ā_2_, Ā_3_, and Ā_4_ are generated. Ā_1_, Ā_2_, Ā_3_, and Ā_4_ are matched together to produce the complete 360° grape model P_0_ shown in Figure 7A. The registered point cloud produces a ghost phenomenon, as shown in Figure 7B. To obtain an accurate grape point cloud model, the normal estimation method, that is, the moving least squares (MLS) method, is used to smooth and resample the point cloud. The voxel grid filter is used to downsample the point cloud model and to solve the minimum values of *N* and *D* in the objective function, as shown in Equations 3, 4. This eliminates the ghost phenomenon of the point cloud model after ICP registration and obtains the accurate normal vector *n* to smooth the whole grape point cloud model, as shown in Figure 7C.


(3) $$\begin{array}{l} H=\min\overset{N}{\underset{i=1}{\sum }}{\left(<n,p_{i}>-D\right)}^{2}\theta \left(\left||p_{i}-q|\right|\right) \end{array}$$

(4) $$\begin{array}{l} \theta \left(d\right)=e^{-\frac{d^{2}}{h^{2}}} \end{array}$$

*p*_*i*_ ∈ *P*_0_*i* = 1, 2, ..., *N*; *D* is the distance from the original point to the fitting plane; *H* is the local reference plane; *n* is the normal vector of the fitting plane; *p*_*k*_ is a point in space; *q* is the projection of *p*_*k*_ on the local reference plane; θ is a smooth decrease function, that is, the weight of each point; *h* represents the smoothing parameter.

### Estimation of Grape Size Characteristics

**Figure 8:**
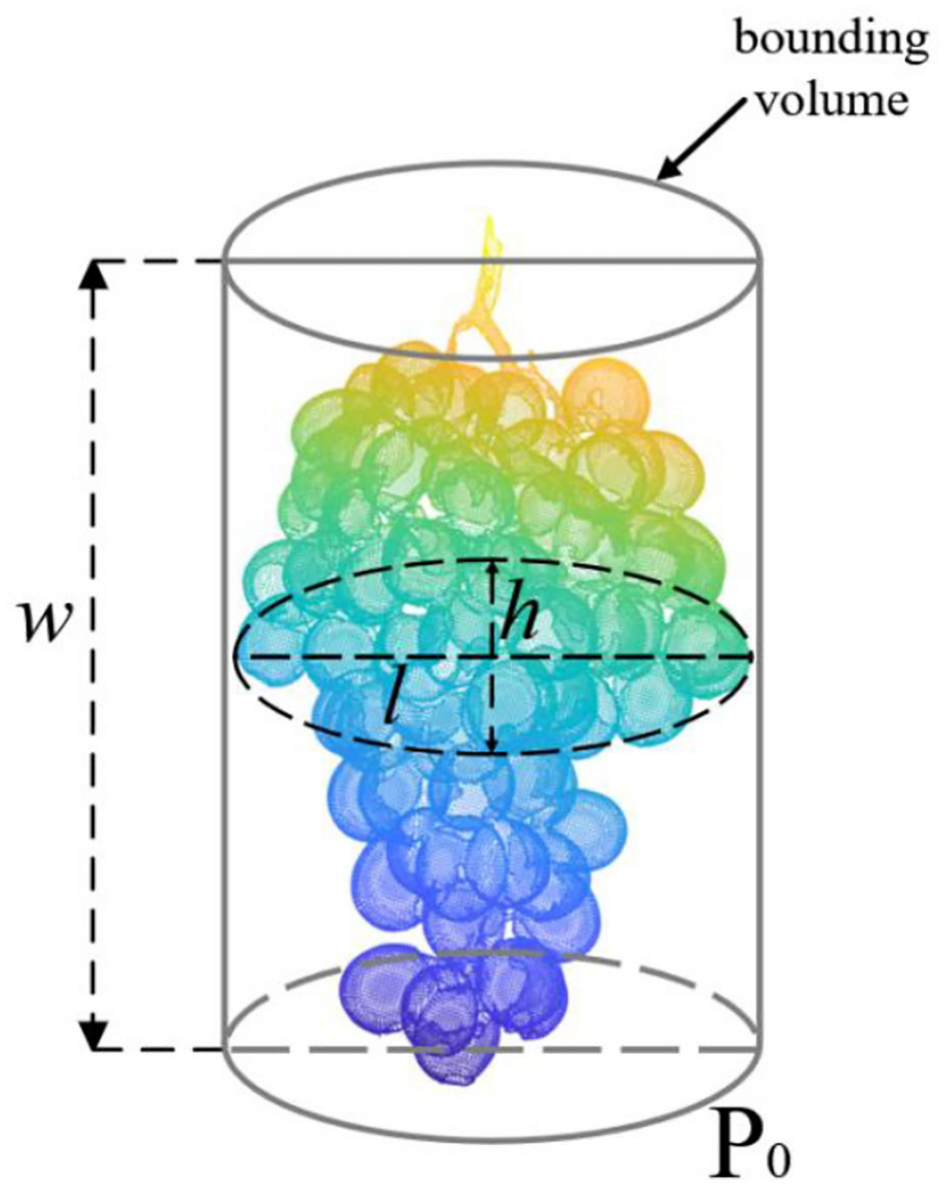
Schematic diagram of the length, height, and width measurement parameters of the grape model. The gray cylinder is a geometric bounding of the grape model.

The length (*l*), width (*w*), and height (*h*) of grape bunches are all characteristic parameters of grapes, which directly affect the evaluation of maturity, quality, and grading and can also reflect the size, growth cycle, and yield. After obtaining an accurate 3D point cloud model, it is specified that the distance along the X-axis corresponds to the length, the distance along the Y-axis corresponds to the width, and the distance along the Z-axis corresponds to the height according to the coordinate system of the grape itself (PCA coordinate system). The calculation steps of fruit length, height, and width are as follows: search all points along the x, y, and z directions; obtain the minimum and maximum values in the x, y, and z directions; and combine the minimum values as *MIN*(*x*_min_, *y*_min_, *z*_min_) and the maximum values as *MAX*(*x*_max_, *y*_max_, *z*_max_). By calculating the Euclidean distance between the minimum value and the maximum value in each direction, the dimensions of the length, width, and height in the grape coordinate system can be obtained, as shown in Equation 5.


(5) $$\begin{array}{l} \{\begin{array}{c} l=x_{\max}-x_{\min}\\ w=y_{\max}-y_{\min}\\ h=z_{\max}-z_{\min} \end{array} \end{array}$$

where *x*_max_ and *x*_min_ are the maximum and minimum values of the x-axis, *y*_max_ and *y*_min_ are the maximum and minimum values of the y-axis, and *z*_max_ and *z*_min_ are the maximum and minimum values of the z-axis, in the PCA coordinate system, respectively. The three-dimensional plane is shown in Figure 8.

### Surface Reconstruction and Volume Estimation

**Figure 9:**
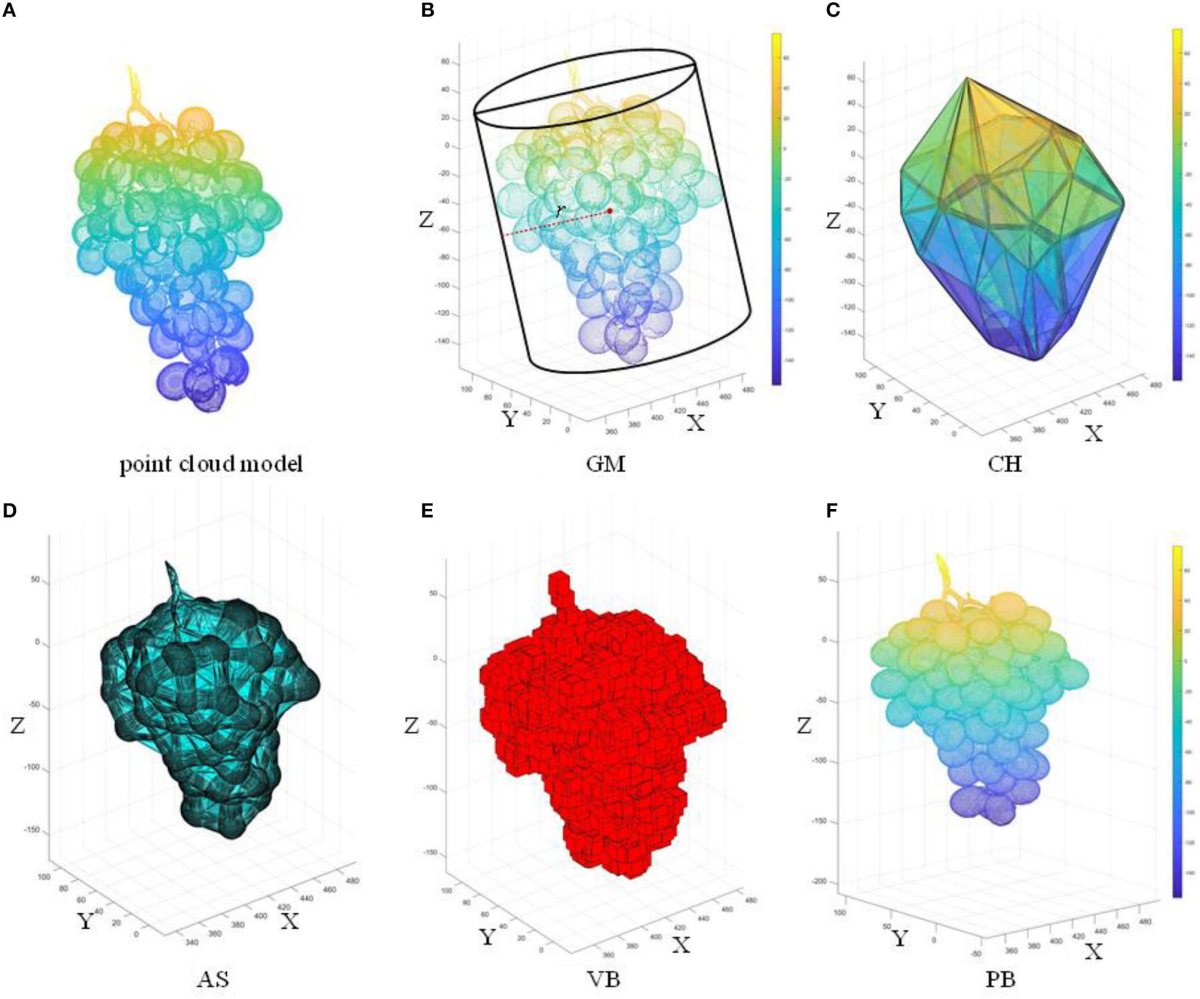
Five surface reconstruction algorithms based on the complete grape point cloud model; **(A)** original point cloud; **(B)** GM; **(C)** CH; **(D)** AS; **(E)** VB; **(F)** PB. *l, w*, and *h* are the three dimensions of the grape (length, width, and height, respectively).

In this section, we show the reconstruction effect on (Figure 9A) of four existing methods: geometric model (GM), 3D convex hull (CH), 3D alpha-shape (AS), and voxel-based (VB) models. The algorithm proposed in this paper [Poisson Reconstruction (PB)] is compared with these methods.

#### Water Displacement Method to Measure the Volume

The grape structure is complex. The different electronic systems have not been considered to detect its volume (Moreda et al., 2005). The Water Displacement Method (WDM) used in various fields is an obvious alternative method (Greenspan et al., 1994; Moreda et al., 2009; Concha-Meyer et al., 2018). It can be advantageous to measure the actual volume of 16 sample grapes using the WDM method.

In this method, the grape cluster is immersed in a beaker filled with water. The drained water is collected in a measuring cylinder so that the volume of the grape cluster can be calculated using the drained water (Marinello et al., 2016; Mohsenin, 2020). The WDM method will be implemented three times, and the average of these three times is taken as the true value of the measured volume of the grape cluster. Because the water is incompressible, the amount of water absorbed by the grapes is very small, and the water temperature is close to the sample temperature. The average of three WDM measurements for each sample can be used to determine the true volume of the sample (Lang and Thorpe, 1989).

#### Reconstruction of the Grape Surface by GM, CH, AS, and VB

A cylinder is one of the simplest regular bodies in three-dimensional space. Many researchers use a simple regular body instead of an irregular body and calculate the geometric model volume as the volume of the body. Therefore, the cylindrical model was used to represent grapes in this study, as shown in Figure 9B. Take the X-axis and Y-axis as the bottom surface. The larger width (w) or length (l) is taken as the diameter 2r. Take the Z-axis as the height h of the cylinder. Volume Vc is given by,


(6) $$\begin{array}{l} V_{c}=\pi r^{2}h \end{array}$$

The shape of the convex hull in the grape is the smallest convex shape that completely contains the grape point cloud *P*_0_, that is, the convex hull of the grape point cloud *P*_0_ in the three-dimensional space is defined by the convex hull surface composed of n vertices in the point cloud *P*_0_. This study used the fast convex hull algorithm (Barber et al., 1996), as shown in Figure 9C. Due to the gaps between the grapes, the bunch has an irregular geometric shape. The representative information of the grapes reconstructed by the convex hull algorithm using the triangular mesh is too rough. The alpha-shape algorithm is a concave hull algorithm (Edelsbrunner et al., 1983), as shown in Figure 9D. Three points are selected from *P*_0_ to form a sphere with radius α, and all points in *P*_0_ are traversed to obtain the triangular mesh model of the grape surface. The parameter α is used to control the fineness of the reconstructed grape representation information. The voxel-based method (Ashburner and Friston, 2000) uses multiple stacked cubes to represent the geometric form of the point cloud, and a voxel contains different numbers of point clouds. The number of cubes containing the grape point cloud can be calculated by counting the number of cubes and the volume of each unit cube, as shown in Equation 7. The results show that the smaller the voxel size is, the more precise the variation of the geometric information of the grape surface can be expressed. According to the specific parameters of the size of the grapes, the length of a single voxel is 0.2 m; the effect of the voxel-based method is shown in Figure 9E.


(7) $$\begin{array}{l} V_{vb}=\overset{n}{\underset{i=1}{\sum }}V_{i}=\overset{n}{\underset{i=1}{\sum }}k_{i}^{3} \end{array}$$

where *V*_*i*_ is the unit cube volume, *K*_*i*_ is the unit cube length, and *V*_*vb*_ is the voxel-based grape volume.

#### Poisson Reconstruction Algorithm

This paper proposes the Poisson reconstruction algorithm (Kazhdan et al., 2006) for the construction of a more accurate object surface, as shown in Figure 9F.

The gradient of the indicator function χ_*P*_0__ is derived from the integral of the surface normal in the grape model. First, use the MLS method in Section Point Cloud Registration to obtain the normal vector of the P_0_. The normal vector *n* is inward. Since the gradient field of the indicator function cannot be calculated, a Gaussian filter is introduced to convolve χ_*P*_0__. It can be proved by the Gaussian divergence theorem that the smoothed surface normal vector field $\int_{\partial P_{0}}{\tilde{F}}_{p_{j}}(p_{i})n(p_{j})dp_{j}$ is equal to the smoothed gradient field $\nabla \left(\chi_{P_{0}}*\tilde{F}\right)\left(p_{i}\right)$ of the indicator function, as shown in the following Equation 8:


(8) $$\begin{array}{l} \begin{array}{c} \nabla (\chi_{P_{0}}*\tilde{F})(p_{i})=\int_{\partial P_{0}}{\tilde{F}}_{p_{j}}(p_{i})n(p_{j})dp_{j} \end{array} \end{array}$$

That is, the surface of P_0_ is ∂*P*_0_, $\tilde{F}$ is the Gaussian filter, ${\tilde{F}}_{p_{j}}\left(p_{i}\right)=\tilde{F}\left(p_{i}-p_{j}\right)$ is the translation of *p*_*i*_ to *p*_*j*_, and *n*(*p*_*j*_) is the normal vector of point *p*_*j*_.

Divide ∂*P*_0_ into different patches *P*_*s*_. The integral over slice *P*_*s*_ is approximated by the value at *s.p* to obtain the vector field $\vec{V}$, as follows Equation 9:


(9) $$\begin{array}{l} \nabla \left(\chi_{P_{0}}*\tilde{F}\right)\left(p_{i}\right)\approx \underset{s\in {\text{P}}_{0}}{\sum }|p_{s}|{\tilde{F}}_{s.p}\left(p_{i}\right)s.n\equiv \vec{V}\left(p_{i}\right) \end{array}$$

where *s.p* is a point sample and *s.n* is the normal on the patch.

A divergence operator is introduced for estimating the indicator function $\tilde{x}$ to form the Poisson equation, as in Equation 10. Solve the $\tilde{x}$ using the Laplace matrix such that it minimizes the distance on space F_o_ between the projection of $\Delta \tilde{x}$ and the projection of $\nabla \cdot \vec{V}$. The model isosurface is then extracted according to $\tilde{x}$ and used to calculate the triangular mesh surface.


(10) $$\begin{array}{l} \Delta \tilde{\chi }=\nabla \cdot \vec{V} \end{array}$$

**Figure 10:**
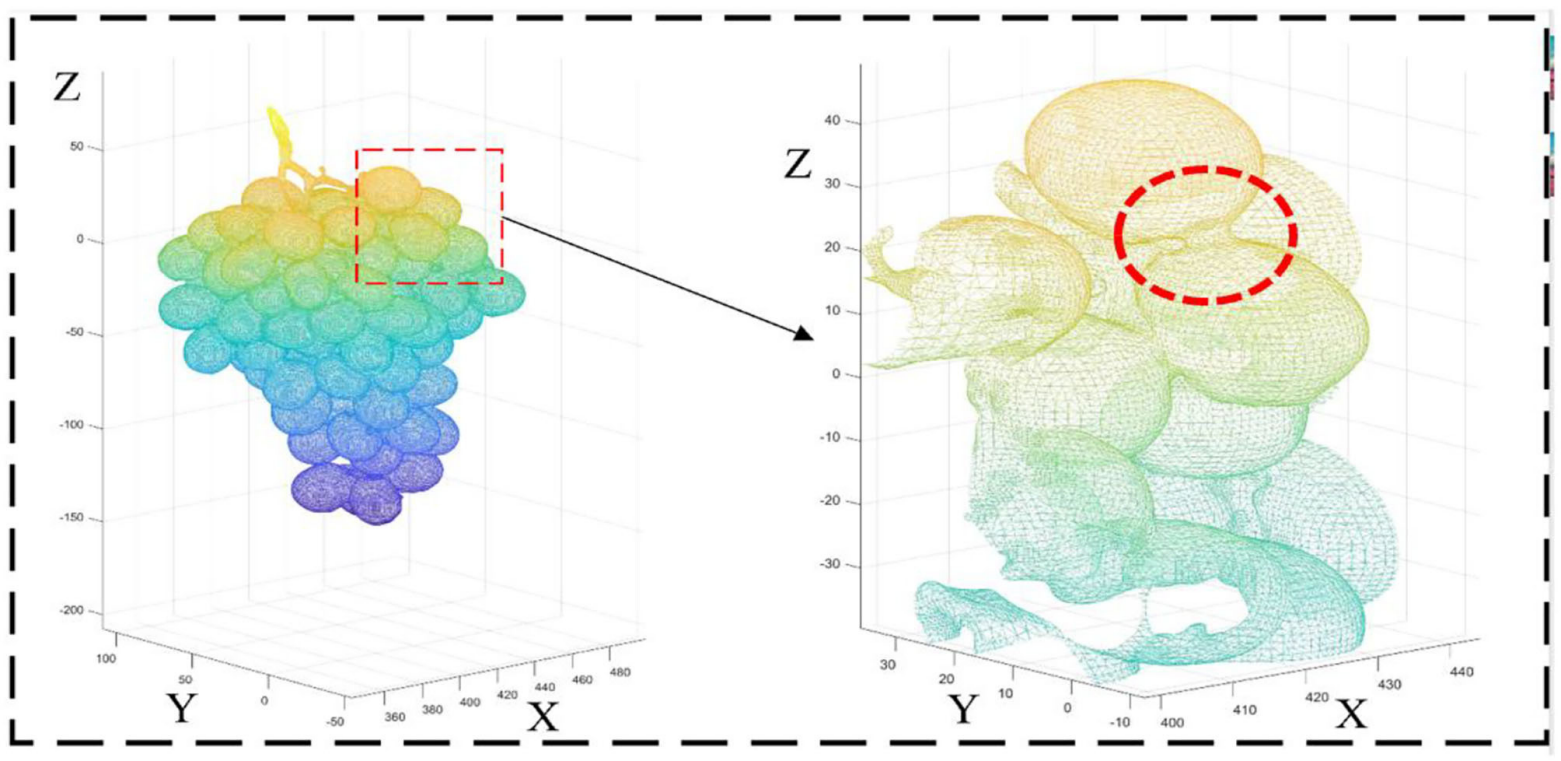
Details of the Poisson reconstruction of the grape surface.

The grape model obtained by Poisson reconstruction is continuous and watertight, which improves the accuracy of volume calculation. Figure 10 shows the details of the Poisson reconstruction of the grape surface.

The volume calculation method of the triangular mesh model (Zhang and Chen, 2001) is to calculate the volume of the tetrahedron corresponding to each triangular face and accumulate the volumes of all tetrahedra in the grape model to obtain the overall *V*_*p*_, where *m* is the number of triangular meshes in the model. v_1_, v_2_, and v_3_ are the three vertices of the triangular face. As in Equation 11, the 3D convex hull and the 3D alpha-shape are used to derive the volume.


(11) $$\begin{array}{l} V_{p}=\overset{m}{\underset{i=1}{\sum }}\frac{1}{6}\left(v_{1}\times v_{2}\right)\cdot v_{3} \end{array}$$

### Model Accuracy Evaluation

In regression tasks, common evaluation metrics are the error (E_*k*_), root mean square error (RMSE), and coefficient of determination (*R*^2^). The RMSE is the square root of the ratio of the sum of the squares of the deviation between the predicted value and the actual value and the ratio of the number of predictions *m*, which is used to measure the deviation between the predicted value and the real value, as shown in Equation 12. The *R*^2^ is used to evaluate the degree of agreement between the predicted value and the true value in the regression model. The numerator part represents the sum of the squared differences between the true and predicted values. The denominator part represents the sum of the squared differences between the true value and the mean of the true value, as in Equation 13. The error formula is shown in Equation 14.


(12) $$\begin{array}{l} \;RMSE\;=\sqrt{\frac{1}{m}\overset{m}{\underset{i=1}{\sum }}{\left(y_{i}-{ŷ}_{i}\right)}^{2}} \end{array}$$

(13) $$\begin{array}{l} R^{2}=1-\frac{\overset{m}{\underset{i=1}{\sum }}{\left(y_{i}-{ŷ}_{i}\right)}^{2}}{\overset{m}{\underset{i=1}{\sum }}{\left(y_{i}-ȳ\right)}^{2}}\in \left[0,1\right] \end{array}$$

(14) $$\begin{array}{l} E_{k}=y_{i}-{ŷ}_{i} \end{array}$$

where *m* is the number of predictions; *y*_*i*_ is the true value; ${\hat{y}}_{i}$ is the predicted value; *y*_*i*_ is the average of the true values; and *k* represents the length (_*l*_), width (_*w*_), and height (_*h*_).

## Discussion and Results

### Comparison of ICP Registration and Modification Methods

**Figure 11:**
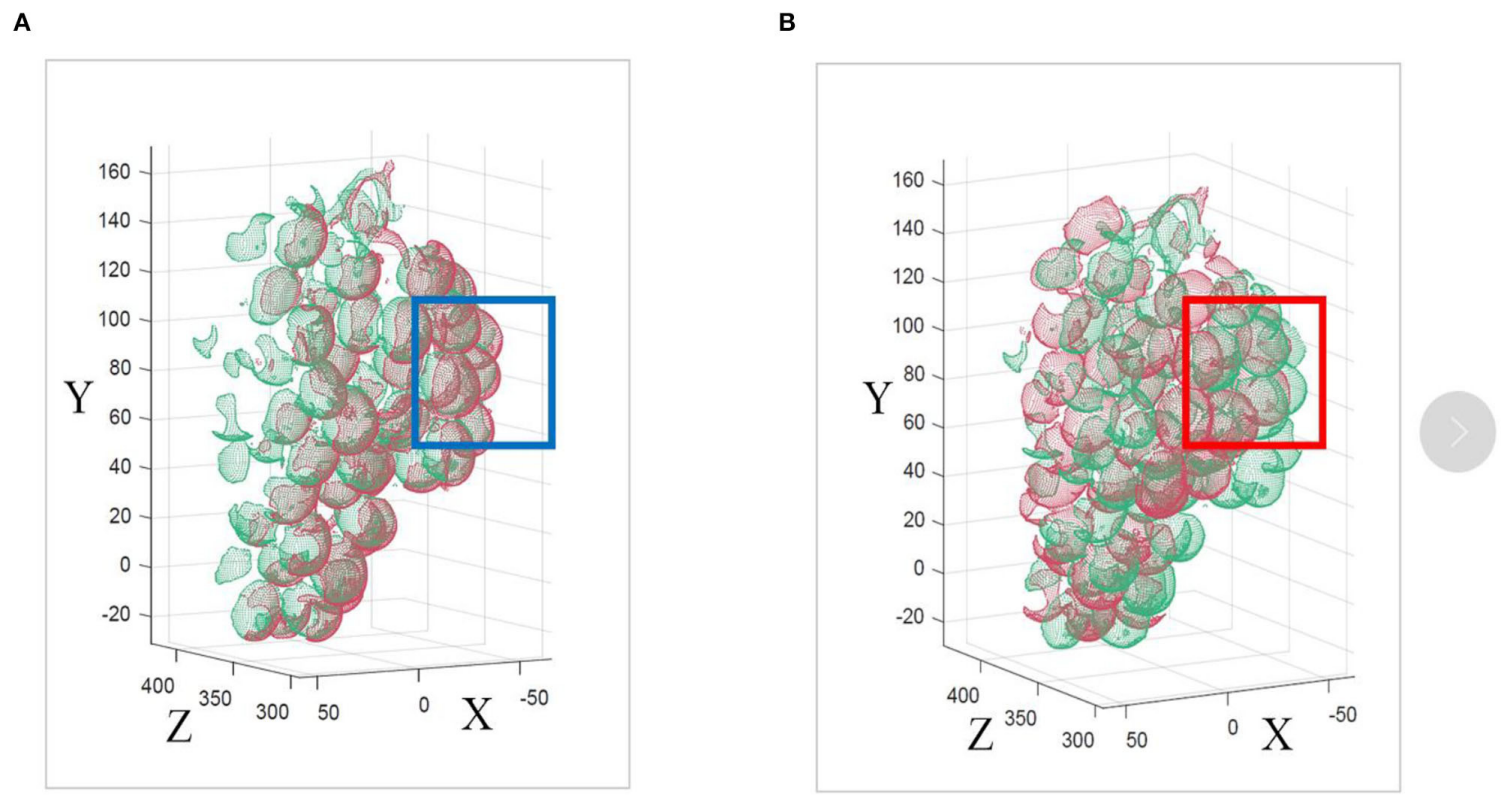
**(A)** Improved ICP algorithm and **(B)** traditional ICP algorithm.

The correct registration of ICP directly affects the accuracy of the entire model, and the selection of the initial pose of the source point set and the point set to be matched directly affects the final matching result of the ICP algorithm. The traditional ICP algorithm cannot judge the pose relationship between the source point set and the point set to be matched, as shown by the blue box in Figure 11B. This paper finds the PCA coordinate system of each point cloud set before ICP registration. This allows the point cloud to be matched to a more reasonable position, as shown by the blue box in Figure 11A. Compared with the traditional ICP algorithm, the improved method can register the point cloud more accurately.

### Effect of Alpha Value on AS

**Figure 12:**
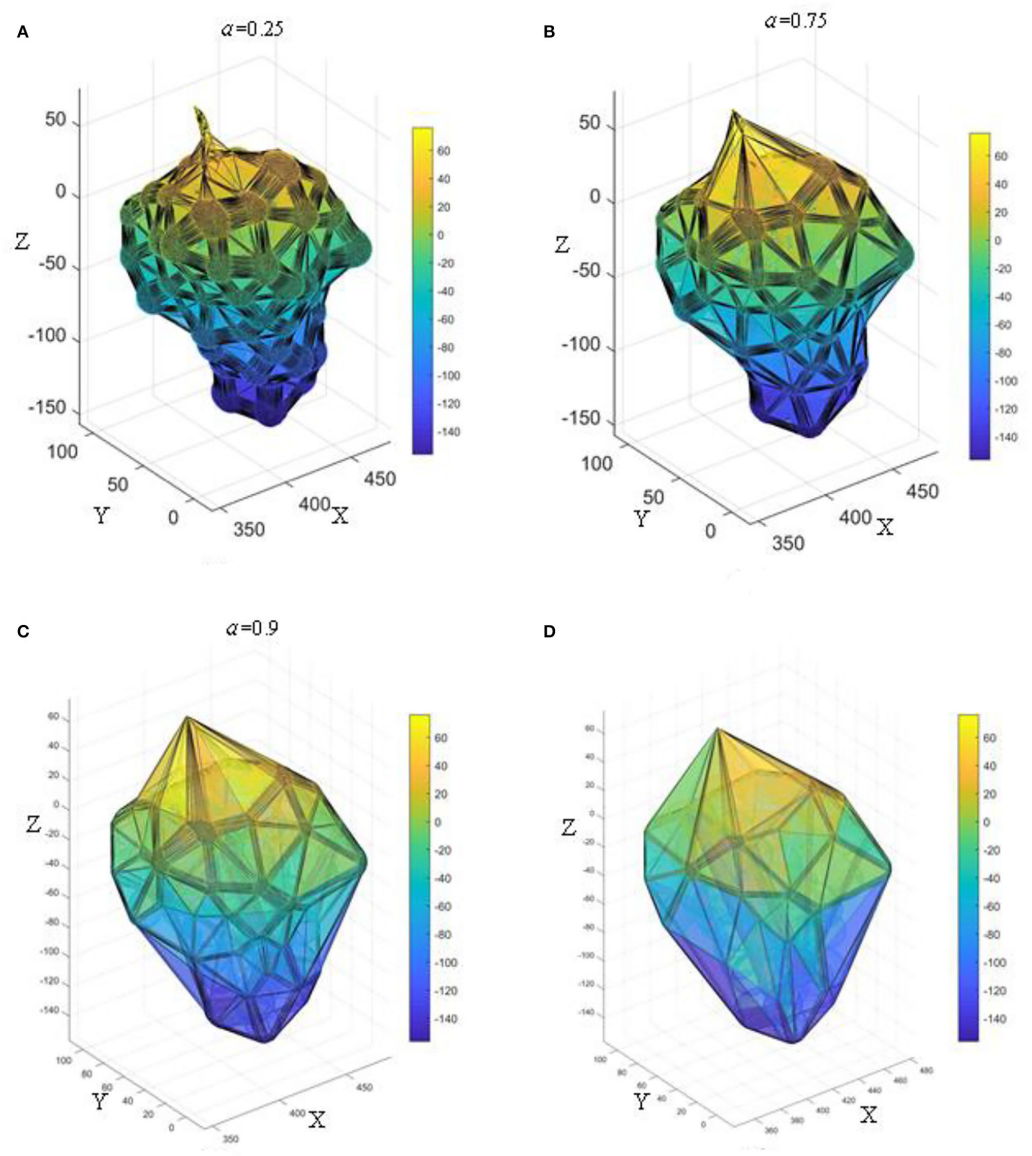
The effect of different α-values on the reconstruction of 3D α-shape; **(A)** α = 0.25; **(B)** α = 0.75; **(C)** α = 0.9; **(D)** 3D convex hull.

It is found that different alpha values greatly affect the reconstruction accuracy of AS. Taking grapes with dense fruit as an example, its point cloud contains a lot of information. Figure 12 shows the effect of grape bunch reconstruction at different alpha values. When α = 0.25, the reconstructed grape characterization information is relatively fine, which can represent its geometric structure and the degree of compactness between grapes, as shown in Figure 12A. When α = 0.75, the pores between the grapes are surrounded by triangular faces, and only the general spatial structure of the grape bunch is reconstructed, but there is no detailed characterization information of the grapes, as shown in Figure 12B. When α = 0.9, the grapes are completely wrapped, the convex hull of the external point cloud is generated, and the information in the internal structure cannot be expressed, as shown in Figure 12C. According to the above analysis, by setting different α values, the grape triangular grid structure with different degrees of fineness can be reconstructed to reflect different spatial shapes, densities, and fruit characteristics. Comparing the renderings generated from different values of α and the CH method (Figure 12D) demonstrates that the grapes reconstructed by the AS have similar effects to those reconstructed by the CH when α is >0.9. The alpha value was set to 0.25 in this study.

### Evaluation of the Contribution of the Pre-built Map

The characteristic parameters of the 16 grape samples measured in real-time were compared with the characteristic parameters estimated by the point cloud to verify the accuracy of the algorithm when it was applied to grape bunches. The results show a discrepancy between estimated and measured values. The real volume is between 583.57 and 862.94 cm^3^, the estimated volume of GM is between 1,837.55 and 4,483.76 cm^3^, the estimated volume of CH is between 1,248.60 and 1,925.40 cm^3^, the estimated volume of AS is between 516.18 and 1,130.54 cm^3^, the estimated volume of VB is between 841.70 and 1,597.80 cm^3^, and the PB estimated volume is between 636.43 and 934.18 cm^3^. *E*_*l*_ is the error between the measured length and the estimated length, *E*_*w*_ is the error of the width, and *E*_*h*_ is the error of the height. The results are detailed in Table 1.

**Table 1:** Size error and volume estimated of the grape sample.

**Sample number**	***E_***l***_/*cm**	***E_***w***_/*c*m***	***E_***h***_/*c*m***	**Volume/cm** ^**3**^					
				**True**	**GM**	**CH**	**AS**	**VB**	**PB**
1	0.24	0.02	−0.39	583.57	2,470.26	1,478.09	652.76	1,031.63	643.75
2	0.19	0.17	0.09	825.26	4,183.38	1,642.80	1,006.54	966.07	881.53
3	0.01	0.13	0.22	599.71	3,226.55	1,480.40	653.73	1,188.59	649.34
4	0.16	0.33	0.21	606.27	3,228.54	1,476.01	788.70	1,220.57	673.46
5	−0.28	0.39	−0.32	764.41	3,602.84	1,581.10	1,073.00	1,106.77	817.86
6	0.15	0	0.06	657.79	1,837.55	1,379.45	844.41	1,124.53	719.26
7	−0.03	0.26	0.04	751.52	3,735.58	1,673.22	932.01	1,011.26	803.42
8	0.12	−0.13	0.05	642.60	3,620.73	1,434.24	652.15	954.61	711.38
9	0.33	−0.10	0.22	738.57	3,905.98	1,602.23	894.01	1,420.82	779.482
10	0.05	−0.11	0.03	862.75	4,483.76	1,925.40	1,130.54	1,597.80	934.18
11	−0.23	0.17	0.05	649.79	3,521.60	1,373.24	871.27	1,182.27	709.47
12	−0.30	0.11	0.02	588.59	2,192.16	1,248.59	516.18	843.26	636.43
13	−0.17	0.16	0.36	666.62	3,677.26	1,631.06	921.38	1,229.84	725.49
14	0.03	0.15	−0.12	862.94	4,298.38	1,808.92	986.29	1,410.73	918.53
15	0.24	0.35	0.33	619.21	3,333.75	1,614.49	732.54	1,037.84	681.97
16	0.30	0.03	0	619.14	2,288.87	1,248.59	716.18	841.70	662.74
*Max*	0.33	0.39	0.05	862.94	4,483.76	1,925.40	1,130.54	1,597.80	934.18
*Min*	−0.30	−0.13	0.36	583.57	1,837.55	1,248.60	516.18	841.70	636.43
*Mean*	0.05	0.12	−0.39	689.92	3,350.45	1,537.36	835.73	1,135.42	746.77

*E_l_ is the error between the measured length and the estimated length, E_w_ is the error of the width, E_h_ is the error of the height; True denotes the measured volume; GM, geometric model; CH, 3D convex hull; AS, 3D alpha shape; VB, voxel-based; PB, Poisson reconstruction algorithm*.

#### Comparative Analysis of Size Estimation

**Figure 13:**
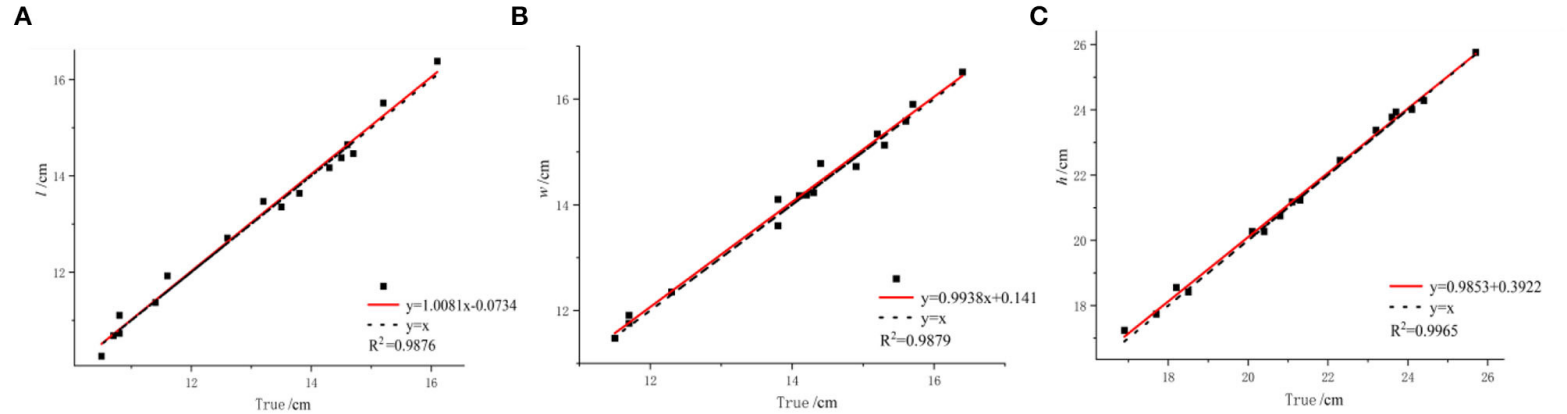
Analysis of estimated grape bunch size. **(A)** Comparison of measured and estimated lengths. **(B)** Comparison of measured and estimated widths. **(C)** Comparison of measured and estimated heights.

Grape size feature detection is an important part of this paper. To evaluate the accuracy of the estimated size, we evaluated the accuracy of the estimate based on the coefficient of determination and root mean square error between the estimated and measured sizes. There is a good correlation between the estimated length and width and the measured length and width. The *R*^2^ value of 0.9876 for length and the *R*^2^ value of 0.9879 for width can be seen as shown in Figures 13A,B, respectively. Some of the estimated length and width values are larger than the measured value and some are smaller than the measured value. As shown in Figure 13C, the *R*^2^ value between the estimated height and the measured height is 0.9965. Most estimated heights are smaller than the measured heights. Due to the accumulation of errors from scanning and the occlusion of the point clouds at the bottom and top of the grape cluster, some details were lost.

Although some estimates fluctuate above and below the measured value, they are within a reasonable range. The estimated size is close to the measured value, especially on grape bunches with few overlapping grapes. The above results verify the accuracy and practicality of the proposed method, which can provide a more accurate basis for the volume calculation.

#### Comparison of the Volume Calculated by Five Methods

The accuracies of the proposed method and four existing methods in volume estimation were determined. The results of the WDM were used as the “true value” for volume to compare against the results of all methods (GM, CH, VB, AS, and PB). We evaluate the impact of different methods on volumetric accuracy by computing the difference between the true value and the model estimate based on linear regression. By analyzing Table 1, it can be concluded that the estimated volumes predicted by GM, CH, and VB are larger than that predicted by AS and PB. Compared with the measured average values, their average values are 2,660.53, 847.44, and 445.5 cm^3^ larger, respectively. In contrast, the volume predicted by AS and PB is closer to the measured value.

**Figure 14:**
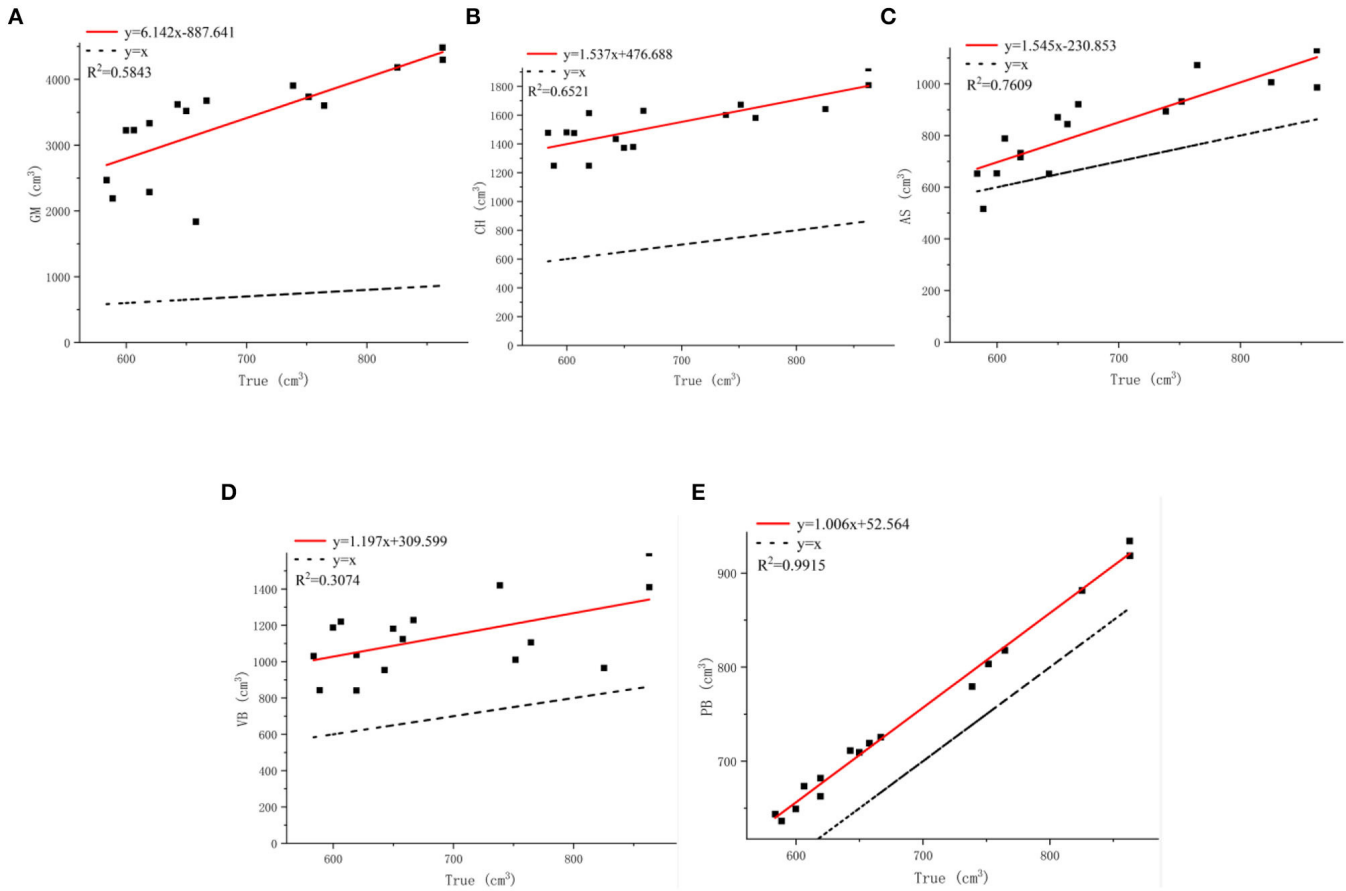
Analysis of grape estimators. **(A)** Comparison of true and GM. **(B)** Comparison of true t and CH. **(C)** Comparison of true and AS. **(D)** Comparison of true and VB. **(E)** Comparison of true and PB.

The GM method replaces the point cloud model with a cylinder of the same width as the model. In Figure 9B, GM considers not only the holes between the grapes as part of the volume but also the space between the point cloud model and the cylinder, which can result in excessive results and large estimation errors. Therefore, the estimated value of GM is much greater than the true value with *R*^2^ = 0.5843 as shown in Figure 14A. The increase in *R*^2^ of the linear fit of the CH method means that the estimated value is more accurate than the GM method, which has an *R*^2^ value of 0.6521 as shown in Figure 14B. From the *R*^2^ value fitted between the measured value of the CH method and the true value, it can be seen that the measured volume change is related to the morphological structure of grapes. Moreover, the CH model is considered as geometry without holes, so the estimated volume is larger. The AS method treats the main point cloud as a whole when α is small enough and outliers are removed. The minimum concave hull is established from the original point cloud as shown in Figure 9E. It can be seen from Figure 14C that AS has an *R*^2^ value of 0.7609 which shows a good correlation between its measured values and the true values. However, the AS method does not handle the subtleties enough to form a 3D model that more closely matched the actual shape of the grape cluster. Locally sparse and missing point clouds can no longer be well-represented in reconstruction. As shown in Figure 9D, The VB algorithm divides the entire grape cluster into several sub-parts, takes part of the point cloud as the calculation object, and uses a fixed-size voxel to describe the volume of the grape. However, in Figure 14D, the estimated value of VB is in low agreement with the true value, and the *R*^2^ value of which is only 0.3074.

The PB method is based on the normal vector fitting. As can be seen from Figure 10, this implicit fitting can produce a smooth surface, which does not introduce additional errors compared to AS, and is more robust to noise. When the density of the collected point cloud is not uniform, there will be no errors such as the transition zone of the space object. The edges of the model are finer than other methods, which can describe the contour information of the grape surface more accurately and characterize the holes and gaps. From Table 1, it can be found that the volume difference is positively correlated with the accuracy of grape surface contour reconstruction, and our method estimates that the grape volume is less different from the true value. Considering the relationship between the accurate quantitative estimates and the true value, we perform a fitting analysis on the volume calculated by PB. In Figure 14E, the *R*^2^ value of the PB method is 0.9915 close to 1, which shows that our method produces good results for the estimation of grape volume. However, considering that the reconstructed surface is watertight, there will be areas of adhesion between the berries as shown by the red circles in Figure 10. Moreover, the camera cannot scan the internal structure of the grape bunch, and its interior will be regarded as a completely closed shape after the reconstruction. These conditions can affect the volume estimation resulting in the estimated values being slightly larger than the true values.

The DM, CH, and VB methods replace the actual grape shape with envelope models resulting in an external space between these models and the actual shape of the grape bunch. Therefore, the calculated volume is much larger than the true value. The AS method can solve this problem to a certain extent; however, the it cannot describe the holes between grapes smoothly. Therefore, the volume calculated by the AS method is less accurate than our method. The proposed Poisson reconstruction method can describe the surface hole information well. However, the method cannot describe the unknown area inside the grape bunch. By maintaining a balance between computational complexity and estimation robustness, our method can estimate the characteristic parameters of the grape bunch relatively accurately.

## Conclusion

This study proposes a point cloud data-based method for estimating characteristic parameters of a single grape bunch. A complete three-dimensional point cloud model of the grape bunch is established. The information on length, width, and height of the point cloud is estimated. Experiments show that the proposed Poisson reconstruction method is the optimal volume estimation algorithm compared with the existing four reconstruction algorithms. Some conclusions are summarized as follows:

(1) By using the point cloud camera and rotating the bunch at intervals, the point cloud of the grape bunch could be completely collected. The point cloud information of more perspectives could be obtained by reducing the angle of each rotation. The target point cloud could be segmented from the collected point cloud information by filtering and region growing algorithm. The point cloud coordinates of each angle could be corrected by using the PCA, and the coarsely matched point cloud could be registered by using the ICP algorithm. The quality of the grape point cloud model could be improved by implementing the MLS algorithm.
(2) The length and height information of the grape bunch could be extracted according to the coordinate system of the grape bunch. The R^2^ values of length, width, and height were all between 0.85 and 0.88 by comparing the correlation between the size estimated by the proposed method and the size measured.
(3) The Poisson reconstruction method could use a smaller triangular mesh to smoothly describe the grape surface. It is more robust to the point cloud with uneven density and performs more finely at the hole. The volume of a single grape bunch calculated by the Poisson reconstruction method was closer to the real value than the volumes calculated by the other four methods. However, due to the inability to describe the internal information of grapes, the value calculated by the Poisson reconstruction method was slightly larger than the true value.

## Data Availability Statement

The original contributions presented in the study are included in the article/supplementary material, further inquiries can be directed to the corresponding authors.

## Author Contributions

WL: data curation, investigation, and writing-original draft. DY: writing, experiment, and editing. WC: writing and editing. CW: conceptualization, data curation, methodology, and supervision. LL: methodology and supervision. All authors contributed to the article and approved the submitted version.

## Conflict of Interest

The authors declare that the research was conducted in the absence of any commercial or financial relationships that could be construed as a potential conflict of interest.

## Publisher's Note

All claims expressed in this article are solely those of the authors and do not necessarily represent those of their affiliated organizations, or those of the publisher, the editors and the reviewers. Any product that may be evaluated in this article, or claim that may be made by its manufacturer, is not guaranteed or endorsed by the publisher.
